# Use of kisspeptin to trigger oocyte maturation during *in vitro* fertilisation (IVF) treatment

**DOI:** 10.3389/fendo.2022.972137

**Published:** 2022-09-06

**Authors:** Bhavna Sharma, Kanyada Koysombat, Alexander N. Comninos, Waljit S. Dhillo, Ali Abbara

**Affiliations:** ^1^ Section of Investigative Medicine, Imperial College London, London, United Kingdom; ^2^ Department of Endocrinology, Imperial College Healthcare NHS trust, London, United Kingdom

**Keywords:** IVF, kisspeptin, *in vitro* fertilisation, oocyte, fertility, ovarian hyperstimulation syndrome, OHSS

## Abstract

Infertility is a major global health issue and is associated with significant psychological distress for afflicted couples. *In vitro* fertilisation (IVF) utilises supra-physiological doses of stimulatory hormones to induce the growth of multiple ovarian follicles to enable surgical retrieval of several oocytes for subsequent fertilisation and implantation into the maternal endometrium. The supra-physiological degree of ovarian stimulation can lead to potential risks during IVF treatment, including ovarian hyperstimulation syndrome (OHSS) and multiple pregnancy. The choice of oocyte maturation trigger, such as human chorionic gonadotrophin (hCG) or gonadotrophin releasing hormone agonist (GnRHa), can impact both the efficacy of IVF treatment with a bearing on luteal phase hormonal dynamics and thus the degree of luteal phase support required to maintain optimal pregnancy rates, as well as on safety of treatment with particular respect to the risk of OHSS. Kisspeptin regulates gonadotrophin releasing hormone (GnRH) release and is therefore a key regulator of the hypothalamo-pituitary-gonadal (HPG) axis. Kisspeptin has been shown to be requisite for the occurrence of the physiological ovulatory luteinising hormone (LH) surge. In this review, we discuss the potential use of kisspeptin as a novel trigger of oocyte maturation.

## Introduction

Infertility is defined as the inability to conceive after one year of regular unprotected sexual intercourse, and is estimated to affect between 12-30% of couples (1). The advent of *in vitro* fertilisation (IVF) treatment has provided an effective means of helping couples afflicted by infertility. Louise Brown was the first child born as a result of IVF treatment in 1978 (2). In 2010, Robert Edwards was awarded the Nobel Prize for his work on the development of IVF treatment (3). Globally, IVF treatment has been estimated to have resulted in over 8 million live births over the last 40 years (4).

Many of the processes in current IVF treatment map across to those occurring during the physiological human menstrual cycle, including: follicular development, oocyte maturation/ovulation, fertilisation by sperm to form embryos, and subsequent implantation into the maternal endometrium (5). However, during IVF treatment, supra-physiological doses of stimulatory hormones are required to induce the growth of multiple ovarian follicles to enable surgical retrieval of several eggs, as compared with the 1-2 follicles that develop during the physiological menstrual cycle (6). The supra-physiological degree of stimulation can unfortunately lead to potential risks during IVF treatment, including ovarian hyperstimulation syndrome (OHSS) and multiple pregnancy.

During IVF, a pharmacological dose of a preparation containing follicle stimulating hormone (FSH) is used to induce multi-follicular growth in the ovaries (7). Thereafter, premature ovulation is prevented either by using a competitive gonadotrophin releasing hormone (GnRH) antagonist, or by chronic administration of a GnRH agonist (GnRHa) to down-regulate pituitary GnRH receptors thereby preventing a premature luteinising hormone (LH) surge (5). This allows follicles the time to grow to the required size whilst avoiding ovulation from occurring prematurely. Once 2-3 follicles of 17-18mm are reached, a hormonal inductor (frequently called the ‘trigger’) of oocyte maturation is used to provide the LH-like exposure needed to mature the oocytes and enable their surgical retrieval. Oocyte maturation refers to the process by which oocytes gain competence for fertilisation by sperm as they traverse from the metaphase I to the metaphase II stage of development by extruding half of their genetic material as the polar body (which is used to identify that successful nuclear oocyte maturation has occurred) (8). This process of oocyte maturation is a prelude to ovulation; thus, the trigger helps synchronise the commencement of this process such that surgical retrieval of oocytes is timed to occur once oocytes have matured but prior to ovulation. Typically, transvaginal oocyte retrieval is scheduled to take place at 36 hours after administration of the trigger agent (6).

It is notable that the LH-like exposure provided by the trigger also determines how likely it is for corpora lutea to survive and produce sex-steroids to support the early phase of pregnancy. Luteal phase sex-steroid production, particularly progesterone produced by the corpus luteum, prepares the endometrium for embryo implantation. Thus, the efficacy of oocyte maturation and the ability of oocytes to be retrieved, as well as implantation rates due to luteal phase hormonal characteristics, are influenced by the choice of trigger employed in IVF cycles (5).

The most widely used trigger of oocyte maturation in IVF treatment is human chorionic gonadotrophin (hCG). Due to its long half-life, potent LH receptor activity with prolonged duration of intracellular effect, hCG is recognised to be a major iatrogenic cause of OHSS (5). GnRHa induces a shorter duration of LH exposure with a peak level at 4-6 hrs after administration. However, use of GnRHa is challenged by concerns that this insufficient duration of LH exposure can exacerbate luteal phase deficiency and hamper pregnancy rates (6). Thus, the trigger of oocyte maturation impacts on both the safety of IVF treatment with particular respect to the risk of OHSS, as well as on the efficacy of IVF treatment, with a bearing on luteal phase hormonal dynamics and thus the degree of luteal phase support required to maintain optimal pregnancy rates. In this review, we discuss the potential of the reproductive neuropeptide kisspeptin for use as a novel trigger of oocyte maturation.

## Kisspeptin

Kisspeptin was initially discovered as a metastatic suppressor gene in melanoma cells and thus was originally termed ‘metastin’ (9). However, its role in fertility became apparent in 2003, when it was recognised that loss of function variants in the gene encoding for the kisspeptin receptor (*KISS1R*) result in congenital hypogonadotrophic hypogonadism and a failure of the reproductive endocrine axis (10, 11). Since then, it has been demonstrated that kisspeptin regulates the activity of GnRH neurons in the hypothalamus and thus the downstream hypothalamo-pituitary-gonadal axis (9).

Kisspeptins are a family of arginine phenylalanine amide (RF amide) peptides encoded by the *KISS1* gene located on chromosome 1q32 (9). Various kisspeptin peptides are derived from proteolytic enzyme cleavage of the 145 amino acid prepropeptide to yield kisspeptins of different amino acid lengths denoted by their suffix (e.g., kisspeptin-54 has 54 amino acids). Kisspeptin-54 (KP54) and kisspeptin-10 (KP10) are the two most widely studied peptides, with KP54 having a longer terminal half-life than KP10 (~28 vs ~3 minutes) (9).

Kisspeptin neurons in the hypothalamus play an important role in integrating sex-steroid feedback from the periphery to regulate GnRH secretion (12). It has been demonstrated that kisspeptin signalling is a requisite for the occurrence of the mid-cycle LH surge and resultant ovulation (13). When a kisspeptin neutralising antibody was applied to the preoptic area of the hypothalamus containing GnRH neurons physiological ovulation was inhibited (13). Kisspeptin was also shown to induce ovulation to a similar extent as hCG in a super-ovulated rodent model (14), providing early credence to the potential use of kisspeptin as a trigger of oocyte maturation in women undergoing IVF treatment.

## Kisspeptin as trigger of oocyte maturation

Kisspeptin was first administered to healthy men in 2005 and induced a robust dose-dependent increase in serum LH levels (15). It was subsequently administered to healthy women in 2007 and interestingly, a differential degree of LH rise was induced depending on the phase of the menstrual cycle in which it was administered (16). Indeed, when the same dose of KP54 (0.4 nmol/kg) was given in the preovulatory phase of the cycle, it resulted in LH rises several folds higher than in the follicular or luteal phases, highlighting that the hormonal milieu at the time of administration impacts on the resultant LH response (16). Moreover, the ability of kisspeptin to induce an ovulatory LH rise suggested that it could be used to induce oocyte maturation in women undergoing IVF treatment (16).

In 2014, the first trial evaluating the use of KP54 to induce oocyte maturation was conducted in 53 infertile women undergoing IVF treatment (17). A single subcutaneous bolus of KP54 was administered at doses between 1.6 and 12.8 nmol/kg during a GnRH antagonist co-treated IVF cycle (17). Kisspeptin induced a peak LH rise of ~45 IU/L at ~5 hours following administration, returning to pre-trigger levels at 12-14 hours after kisspeptin (17). The vast majority of women (51 of 53 women) had at least one mature oocyte retrieved and at least one embryo (49 of 53 women) available for implantation (17). Importantly, 12 healthy babies (from 8 singleton and 2 twin pregnancies) were born following kisspeptin triggering in this study (17). This ‘proof of concept’ study first demonstrated that kisspeptin could be used as a trigger of oocyte maturation in women and result in healthy pregnancy. (see **Table 1** for summary of data from three trials evaluating KP54 as a novel trigger of oocyte maturation in IVF).

**Table 1:** Summary of data from three clinical trials evaluating KP54^c^ as a novel trigger of oocyte maturation in IVF treatment.

	Average no. of oocytes Mean (SD)	Average no. of M2 oocytes Mean (SD)	≥1 mature oocyte N (%)	Oocyte yield^a^(%)	≥1 fertilised egg N (%)	Average no. of 2PN zygotes Mean (SD)	Average no. of D3 embryos Mean (SD)	Average no. of D5 embryos Mean (SD)	Average no. of HQ D5 embryos Mean (SD)	Embryo transfer performed N (%)	High-quality embryo transfer performed N (%)	Biochemical pregnancy rate per transfer(%)	Clinical pregnancy rate per transfer(%)	Live birth rate per transfer(%)	Moderate to severe OHSSN (%)
**Jayasena, Abbara et al., 2014** (17) **–** *First phase 2 randomized trial to evaluate the use of KP54^b^ to induce oocyte maturation in 53 women undergoing IVF treatment*															
Kisspeptin dose range 1.6-12.8nmol/kg (n = 53)	9.2 (4.2)	7.8 (3.9)	51/53 (96.2%)	85.1%	49/53 (92.5%)	5.6 (3.5)	5.3 (3.3)	4.1 (3.4)	1.3 (1.6)	49/53 (92.5%)	31/53 (58.5%)	21/49 (42.9%)	12/49 (24.5%)	10/49 (20.4%)	0
**Abbara, Jayasena et al., 2015** (18) **–** *Phase 2, open-label, randomized trial on the use of KP54^b^ to induce oocyte maturation in 60 women at high risk of developing OHSS*															
Kisspeptin dose range 3.2-12.8nmol/kg (n = 60)	14.4 (10.0)	11.2 (8.6)	57/60 (95.0%)	95.1%	54/60 (90.0%)	9.0 (7.2)	8.9 (7.2)	8.4 (7.1)	1.8 (2.4)	51/60 (85.0%)	37/60 (61.7%)	32/51 (62.7%)	27/51 (52.9%)	23/51 (45.1%)	0
**Abbara, Clarke et al., 2017** (19) **–** *Phase 2, randomized, placebo-controlled trial of 62 women at high risk of OHSS on the impact of a second dose of KP54^b^*															
Kisspeptin 9.6nmol/kg + placebo (n = 31) – Single group^c^ or + 9.6nmol/kg (n = 31) – Double group^d^	12.3 (6.1)	9.7 (5.3)	61/62 (98.4%)	68.5%	61/62 (98.4%)	7.2 (4.1)	7.1 (4.0)	5.9 (3.6)	1.6 (2.0)	60/62 (96.8%)	34/62 (54.8%)	26/60 (43.3%)	19/60 (31.7%)	18/60 (30.0%)	1/62 (1.6%)
**All 3 trials –** *Summary of data from three clinical trials evaluating KP54^b^ as a novel inductor of oocyte maturation during IVF treatment*															
n = 175	12.1 (7.6)	9.7 (6.5)	169/175 (96.6%)	82.9%	164/175 (93.7%)	7.3 (5.4)	7.2 (5.4)	6.2 (5.3)	1.6 (2.0)	160/175 (91.4%)	102/175 (58.3%)	79/160 (49.4%)	58/160 (36.3%)	51/160 (31.9%)	1/175 (0.57%)

Data are presented as Means (SD) for continuous variables and Total (Percentages of N) for categorical variables.

^a^Oocyte yield, Proportion of mature oocytes retrieved from follicles of sufficient size (≥14mm) to yield an oocyte.

^b^KP54, Kisspeptin-54.

^c^Single group, All patients received 9.6nmol/kg of KP54 subcutaneously 36h prior to oocyte retrieval to trigger oocyte maturation. Patients randomised to single group received placebo (saline) 10 hours later.

^d^Double group, All patients received 9.6nmol/kg of KP54 subcutaneously 36h prior to oocyte retrieval to trigger oocyte maturation. Patients randomised to double group received KP54 9.6nmol/kg 10 hours later.

## Efficacy of oocyte maturation with kisspeptin

**Figure 1:**
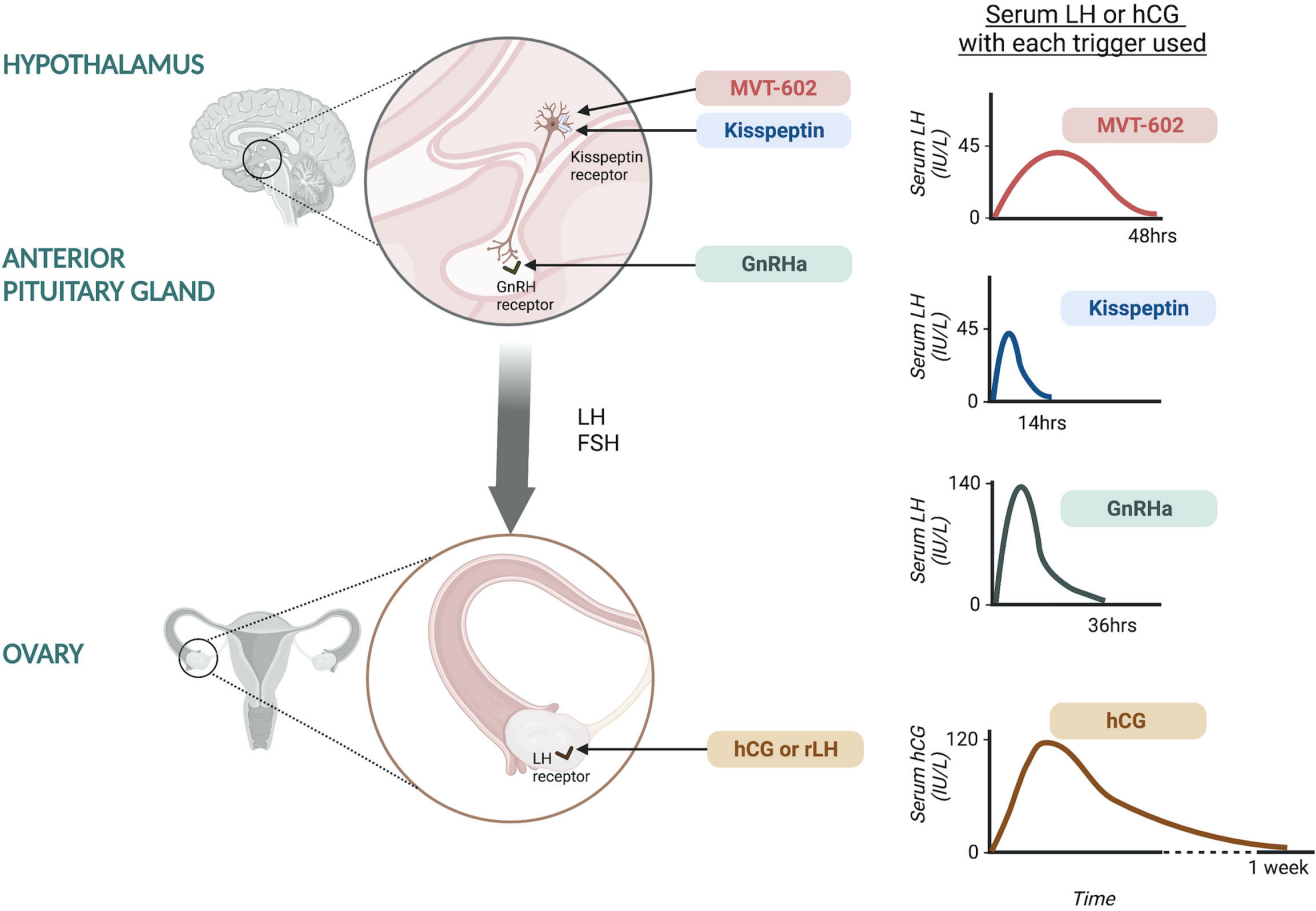
Site of action of oocyte maturation triggers during IVF treatment and the resultant serum LH or hCG response. Kisspeptin and MVT-602 act at the level of the hypothalamus to stimulate kisspeptin receptors on GnRH neurons leading to GnRH release. Kisspeptin induces a peak LH rise of ~45 IU/L at ~5 hours following administration, returning to pre-trigger levels at 12-14 hours. MVT-602 induces a peak of LH rise of similar amplitude to that of the native peptide, KP54, however the duration of the LH rise was markedly prolonged, time of peak LH ~21-22 hours. GnRHa acts at the level of the anterior pituitary on GnRH receptors to stimulate endogenous LH and FSH secretion. GnRHa induces a peak LH level of 140.4 IU/L at ~4 hours after administration. hCG and rLH act at the level of the ovary, directly at the LH receptors. hCG induces a peak hCG level of 121.0 IU/L occurring 24 hours after administration.

The minimum LH-like exposure needed for oocyte maturation during IVF treatment was recently described in 499 IVF cycles triggered with either hCG, GnRHa or kisspeptin (20) (**Figure 1**). As discussed earlier, LH levels after kisspeptin peak at 4-6 hours following administration to reach a peak level of ~45 IU/L (17). The physiological midcycle LH surge has an average amplitude of 56.5 IU/L with a standard deviation of 23.4 (range 25-144 IU/L) (21). Overall, the amplitude of rise in LH after kisspeptin was more similar to that observed after the physiological mid-cycle LH surge, than after GnRHa which induces a peak LH level of 140.4 IU/L at 4 hours after administration (20). Notably, LH levels just prior to trigger administration impacted on the resultant rise in LH (20), highlighting that managing LH levels during ovarian stimulation can modulate the endocrine response to the trigger and thus clinical outcomes.

Peak hCG levels of 121.0 IU/L occurred at 24 hours after administration, however it is more challenging to compare these levels to LH (20). Interestingly, hCG levels negatively correlated with endogenous LH levels (20). It is believed that hCG induces more potent signaling at LH receptors, but with differential intracellular signaling pathways upon activation of the LH receptor (22). Studies of receptor binding kinetics by rotational diffusion demonstrated that LH receptors bound by hCG were immobile, whilst those bound by LH were rotationally mobile, potentially accounting for the differences in receptor activation and subsequent intracellular signaling (22). hCG binds to the LH receptor with higher affinity than LH and is 5-fold more potent in stimulating cAMP activity and has greater steroidogenic action than LH. Whilst LH binding is associated with greater effect on the extracellular signal-related kinase 1/2 and protein kinase B which are anti-apoptotic proliferative signals (22). This is postulated to relate to the different physiological role of hCG and LH, whereby LH is key in oocyte maturation and ovulation, whilst hCG supports the developing embryo and decidua through steroidogenesis (22).

In order to assess the efficacy of the trigger, it is necessary to identify the size of follicles that should be expected to yield oocytes if appropriate triggering is administered (23). Expressing the number of mature oocytes as proportion of this denominator can be termed the ‘mature oocyte yield’ (23). The odds of achieving median mature oocyte yield across all three triggers were increased by the amount of LH-like activity (>60% of patients achieved at least the medial oocyte yield at an hCG level of 160 IU/L at 24 hours, or an LH level of 50 IU/L at 12 hours after GnRHa, or an LH level >10 IU/L at 12 hours following kisspeptin) (20). Progesterone levels were observed to increment immediately following the administration of the trigger and interestingly was inversely associated with LH rise (20). The rise in progesterone after trigger administration relates closely to the number of mature oocytes generated and was found to be more reliable than LH rise in predicting subsequent oocyte retrieval (20). Notably GnRHa induced a greater progesterone rise per oocyte matured than either kisspeptin or hCG triggers (20).

It was interesting that a five-fold lower LH rise at 12 hours after kisspeptin was sufficient to induce similar oocyte maturation in comparison to GnRHa (20). This could be consistent with suggestions of enhancement of oocyte maturation due to a direct action of kisspeptin at ovarian kisspeptin receptors (24). Indeed, conditional ablation of kisspeptin receptors in mice oocytes caused premature ovulatory failure (25). Human ovaries are known to express both *KISS1* and *KISS1R* (26). The direct action of kisspeptin on the ovary was first described in granulosa lutein (GL) cells isolated from follicular fluid aspirated and pooled from follicles during the retrieval of oocytes for IVF/ICSI (27). 48 women undergoing IVF treatment were included in the study; hCG (n=12), GnRHa (n=12) and kisspeptin (n=24) were used as oocyte maturation triggers (27). Expression of genes involved in ovarian reproductive function and steroidogenesis in GL cells retrieved from different oocyte maturation trigger cycles were compared. The GL cells in the kisspeptin trigger group demonstrated higher expression of FSH and LH/hCG receptor, and higher expression of genes involved in ovarian steroidogenesis: steroid acute regulatory protein, 3β-hydroxysteroid dehydrogenase type 2, aromatase, inhibin A, oestrogen receptors alpha and beta compared to traditional triggers of maturation (27). Additional evidence for a direct effect of kisspeptin on oocyte maturation, comes from data demonstrating that kisspeptin enhances *in vitro* maturation of ovine (28) and porcine (29) immature oocytes. However, it is unlikely to do so alone in the absence of gonadotrophins.

Although the LH amplitude attained is similar, the duration of LH rise induced by KP54 is of shorter duration than that of the midcycle physiological LH surge which is triphasic with a plateau lasting 24 hours (18). In order to assess whether extending the duration of the LH surge could better simulate the physiological LH surge and improve oocyte maturation, a study investigated the impact of a second dose of kisspeptin at 10 hours following the first (19). Women (n=62) at high risk of OHSS received a single dose of KP54 (9.6 nmol/kg) at 36 hours prior to oocyte retrieval, following which half of the women were randomised to receive placebo (saline) at 10 hours following the first dose (single), and half to a second dose of KP54 (9.6 nmol/kg) (double) (19). A higher mature oocyte yield was observed in women randomised to the double group, with the proportion of women achieving an above average mature oocyte yield of ≥60%, increasing from 45% in the single group to 71% in the double group (19). The live birth rate per protocol was non-significantly higher at 39% in the double group (19) (**Table 1**). Importantly, this improvement in oocyte yield with a second dose of kisspeptin was not associated with an increase in OHSS, nor with an increase in the proportion of women with a very high number of eggs retrieved (sometimes described as ovarian hyper-response) (19). Studying the changes in LH, hinted as to why this was the case. The second dose of kisspeptin provided an ‘individualised’ LH response, whereby a further LH rise after the second dose of kisspeptin was only observed in those with a lower LH level at 10 hours following the first dose of kisspeptin (19). Conversely, those with an insufficient LH rise, had a ‘rescue LH’ response. Thus, a second dose was able to regress responses towards the mean by eliminating poor responses without exacerbating the occurrence of an excessive ovarian response (19).

Pregnancy rates following kisspeptin have yet to be directly prospectively compared to other triggers, but appear to be at least comparable to current triggers; live birth rate per embryo transfer following all kisspeptin doses tested (including less effective doses) was 32% (51/160) (5), and up to 45% (23/51) in high responders (18), whereas the contemporaneous live birth rate per transfer in women under 35 years treated with fresh embryo transfer in the UK was 32.8% (30).

Recently, a kisspeptin receptor analogue was shown to induce a longer pharmacodynamic action than the native peptide, KP54. MVT-602, previously known as TAK-448, is a nanopeptide kisspeptin receptor agonist developed through modification of KP-10 to have enhanced stability, potency and water solubility (31). The pharmacokinetic, pharmacodynamic and endocrine profile of MVT-602 was recently described in healthy women as well as those with hypothalamic amenorrhea or polycystic ovary syndrome (PCOS) (32). MVT-602 induced an LH surge of similar amplitude to that of the native peptide, KP54, however the duration of the LH rise was markedly prolonged (time of peak LH: MVT-602 21-22 hours vs KP54 4.7 hours). Thus, overall, this resulted in a more than four-fold increase in the area under the curve of the LH exposure (32). This study was conducted outside of the context of ovarian stimulation, but the amplitude and duration of LH rise induced by MVT-602 is likely to be most similar to the physiological mid-cycle LH-surge if studied in the context of ovarian stimulation. The translational use of MVT-602 as potential oocyte trigger in IVF cycles is thus an exciting area for future research to ascertain whether its profile can lead to improved clinical outcomes.

## Does triggering oocyte maturation with kisspeptin reduce the risk of OHSS?

OHSS is one of the main reasons leading to premature IVF cancellation prior to egg retrieval due to unexpected over-response. In addition to the costs of treatment of OHSS, there is also significant psychological morbidity associated with failed or cancelled IVF cycles. OHSS is predominantly an iatrogenic condition that arises due to the aberrant release of vasoactive substances such as vascular endothelial growth factor (VEGF) from the ovary after excessive ovarian stimulation (33). This causes leakage of fluid from the vascular compartment and third-spacing of fluids, for example into the abdomen leading to ascites, or into the lungs causing pleural effusions (19). Furthermore, this depletes the intravascular compartment resulting in haemoconcentration, an increased risk of renal dysfunction, and thromboembolic events. The incidence of severe OHSS associated with haemoconcentration is estimated at 2-6%, moderate OHSS with ascites in 10% and mild (symptoms alone) in 20-33% of cycles (34). Although modern practices, such as increased use of the GnRH antagonist co-treated protocol, are purported to have reduced the risk of OHSS, a recent randomised controlled trial conducted in the UK between 2016-2019 including 619 women, still reported rates of OHSS in women having a fresh transfer at 8.1% (35). This speaks to the difficulty in accurately predicting ovarian response and avoiding OHSS, whilst hCG remains the most commonly used trigger of oocyte maturation.

Symptoms of OHSS (such as abdominal pain or vomiting) are dismissed as being ‘clinically insignificant’ by some practitioners as they are usually self-limiting, with rates of mild OHSS not always reported in many studies. Diagnosis of ‘clinically significant’ (i.e., moderate or severe) OHSS usually requires ultrasound examination and blood testing which is not routinely conducted. Thus, even though OHSS is a well-recognised clinical entity concerns around under-reporting remain, especially in studies where patients are not routinely screened for OHSS and if diagnosis is not made using objective criteria (36). A phase IV randomised controlled trial published in 2016, randomised 1050 women to the two most widely used IVF protocols to assess OHSS rates (37). Notably, most women reported symptoms of OHSS after treatment including: abdominal distention (54%), abdominal discomfort (85%), abdominal pain (61%), nausea (35%), vomiting (2%), dyspnea (16%) and oliguria (3%) (37).

hCG is the most widely used ‘ovarian trigger’ to induce oocyte maturation. Unfortunately, it is also the most frequent cause of OHSS secondary to its long half-life, potent LH receptor activity and long duration of intracellular effect (5). GnRHa is used as an alternative agent particularly in females deemed ‘high risk for OHSS’ however implantation rates have been reported as lower due to luteal phase insufficiency. A small dose of hCG is sometimes used to rescue the luteal phase insufficiency, however this approach can also increase the rate of OHSS (38).

A randomised controlled study investigated whether the use of kisspeptin as trigger of oocyte maturation could avoid OHSS in 60 women at high *a priori* risk (defined as total antral follicle count (AFC) of >23 or a serum anti-Müllerian hormone (AMH) level ≥40 pmol/L) (18). This study observed a dose-dependent increase in the mature oocyte yield 53% at 3.2 nmol/kg, 86% at both 6.4 nmol/kg and 9.6 nmol/kg, and 121% at 12.8 nmol/kg (18). The live birth rate per transfer was 45%, and importantly without any clinically significant (moderate or severe) OHSS (18). As described previously the addition of a second dose of KP54 in women at high risk of OHSS in a further study of 62 women also did not increase the risk of OHSS (19). Besides its shorter duration of action, some additional actions of kisspeptin have been hypothesized for its ability to avoid OHSS. Kisspeptin has been suggested to have a direct action *via* ovarian kisspeptin receptors to suppress the release of VEGF from the ovary and reduce the risk of OHSS (18, 39).

Although a prospective trial comparing clinical outcomes including OHSS to current triggers has yet to be conducted, a retrospective single center study compared rates of OHSS following hCG (n=40), GnRHa (n=99) or kisspeptin (n=122) in women at high risk of OHSS (defined as AFC or total number of follicles on day of trigger >23) (40). The odds ratio (OR) for developing OHSS was significantly higher following hCG (OR 33.6, 95% confidence interval (CI) 12.6-89.5) or GnRHa (OR 3.6, 95% CI 1.8-7.1) compared to kisspeptin (40). Symptoms of OHSS were also least prevalent after kisspeptin, whilst those administered hCG were most symptomatic. Abdominal pain occurred in 69% following hCG, 22% following GnRHa compared to 12% following kisspeptin (40). Similarly vomiting occurred in 8% following hCG, 4% following GnRHa compared to 1% following kisspeptin (40). Furthermore, objective signs of OHSS such as ovarian volume or ascitic volume were improved after kisspeptin (median ovarian volume: hCG 138ml, GnRHa 73ml, kisspeptin 44ml) (40). Compared to baseline ovarian volume, the fold rise in ovarian volume was 20-fold greater following hCG, 8-fold after GnRHa, and 5-fold following kisspeptin (40). Thus, a prospective study directly and objectively characterising signs and symptoms of OHSS is needed to establish whether kisspeptin does indeed reduce the risk of OHSS during IVF treatment.

## Conclusion and future directions

A significant amount of physician time is dedicated to predicting ovarian response, in order to tailor gonadotrophin dosing to avoid over-response. hCG remains the current first-line trigger of oocyte maturation being used in three quarters of cycles (41), however it carries a risk of OHSS, and is the only trigger option available in GnRH agonist co-treated cycles (5). GnRHa triggering can be used in the context of GnRH-antagonist co-treated cycles, and reduces OHSS risk, but many practitioners elect to employ a freeze-all approach due to the increased difficulty in maintaining pregnancy after fresh embryo transfer (now used in more than one third of cycles conducted in the UK) (30). However, the freeze-all approach also carries an increased risk of large for gestational age babies, maternal pre-eclampsia and postpartum haemorrhage (42, 43). Furthermore, women who are not ‘high responders’ i.e., have fewer than 15 eggs retrieved could be disadvantaged by the freeze-all approach (44). Thus, a trigger than can enable “OHSS-free” fresh transfer regardless of the risk of ovarian hyper-response, with the option of subsequent frozen transfer if needed, would be of value for future clinical practice.

Kisspeptin is part of the physiological mechanism for instigating ovulation (12), and induces LH levels of an amplitude more similar to that of the natural midcycle LH surge than by currently available triggers (20). Kisspeptin receptor agonists are in development (32), which also induce a duration of LH-rise that more closely resembles that of the endogenous LH surge (21), however data on their use in IVF treatment is not yet available. In addition to its predominant mechanism to activate hypothalamic GnRH release (12), kisspeptin may also have a direct ovarian action to enhance oocyte maturation (25–27) and to decrease VEGF secretion and OHSS (39). Direct prospective randomised comparison between kisspeptin and currently available clinical triggers is needed in order to establish the impact on clinical outcomes of the more physiological levels of gonadotrophins observed after kisspeptin, however, retrospective data suggests that rates of OHSS are reduced, and live birth rates are at least comparable. Thus, further clinical trials are required to provide the required evidence for the use of kisspeptin to trigger oocyte maturation and inform future clinical practice.

## Author contributions

BS and KK, researched the material and wrote the first draft. BS and KK, joint first authors. WD and AA, co-corresponding and senior authors. All authors contributed to the article and approved the submitted version.

## Funding

This work was supported by grants from the National Institute of Health Research (NIHR), the NIHR/Wellcome Trust Imperial Clinical Research Facility, and the NIHR Imperial Biomedical Research Centre. The Section of Endocrinology and Investigative Medicine was funded by grants from the Medical Research Council (MRC), Biotechnology and Biological Sciences Research Council (BBSRC), NIHR and was supported by the NIHR Biomedical Research Centre Funding Scheme.

## Conflict of interest

WSD and AA have conducted consulting work for Myovant Sciences Ltd. AC is supported by the National Health Service. WD is supported by an NIHR Senior Investigator Award. AA is supported by an NIHR Clinician Scientist Award (No. CS-2018-18-ST2-002). KK is supported by an NIHR Academic Clinical Fellowship Award (No. ACF-2021-21-001).

The remaining author declares that the research was conducted in the absence of any commercial or financial relationships that could be construed as a potential conflict of interest.

## Publisher’s note

All claims expressed in this article are solely those of the authors and do not necessarily represent those of their affiliated organizations, or those of the publisher, the editors and the reviewers. Any product that may be evaluated in this article, or claim that may be made by its manufacturer, is not guaranteed or endorsed by the publisher.
